# Reduction of Protein Bound Methionine Sulfoxide by a Periplasmic Dimethyl Sulfoxide Reductase

**DOI:** 10.3390/antiox9070616

**Published:** 2020-07-14

**Authors:** Lionel Tarrago, Sandrine Grosse, David Lemaire, Laetitia Faure, Mathilde Tribout, Marina I. Siponen, Mila Kojadinovic-Sirinelli, David Pignol, Pascal Arnoux, Monique Sabaty

**Affiliations:** 1 CEA, CNRS, BIAM, Aix Marseille University, F-13108 Saint Paul Lez Durance, France; sandrine.grosse@cea.fr (S.G.); david.lemaire@cea.fr (D.L.); Laetitia.faure04@gmail.com (L.F.); mathilde_tribout@orange.fr (M.T.); marina.siponen@cea.fr (M.I.S.); Mila.Sirinelli@univ-amu.fr (M.K.-S.); david.pignol@cea.fr (D.P.); pascal.arnoux@cea.fr (P.A.); 2 INRAE, Aix Marseille University, BBF, F-13009 Marseille, France

**Keywords:** dimethyl sulfoxide reductase, enzyme kinetics, methionine sulfoxide, oxidative stress, protein oxidation, protein quality control, *Rhodobacter sphaeroides*

## Abstract

In proteins, methionine (Met) can be oxidized into Met sulfoxide (MetO). The ubiquitous methionine sulfoxide reductases (Msr) A and B are thiol-oxidoreductases reducing MetO. Reversible Met oxidation has a wide range of consequences, from protection against oxidative stress to fine-tuned regulation of protein functions. Bacteria distinguish themselves by the production of molybdenum-containing enzymes reducing MetO, such as the periplasmic MsrP which protects proteins during acute oxidative stress. The versatile dimethyl sulfoxide (DMSO) reductases were shown to reduce the free amino acid MetO, but their ability to reduce MetO within proteins was never evaluated. Here, using model oxidized proteins and peptides, enzymatic and mass spectrometry approaches, we showed that the *Rhodobacter sphaeroides* periplasmic DorA-type DMSO reductase reduces protein bound MetO as efficiently as the free amino acid L-MetO and with catalytic values in the range of those described for the canonical Msrs. The identification of this fourth type of enzyme able to reduce MetO in proteins, conserved across proteobacteria and actinobacteria, suggests that organisms employ enzymatic systems yet undiscovered to regulate protein oxidation states.

## 1. Introduction

Either free or included in proteins, the sulfur containing amino acid methionine (Met) can be oxidized to methionine sulfoxide (MetO) which exists as two diastereomers, methionine-*S*-sulfoxide (Met-*S*-O) and methionine-*R*-sulfoxide (Met-*R*-O) [1]. Formation of MetO in proteins was shown to alter their function in numerous ways, from damageable effects for their activity and stability, to controlled regulation of enzyme activity and protein-protein interactions [2,3,4,5,6]. Contrary to oxidative modifications of most other amino acids, the oxidation of Met into MetO in proteins is reversible thanks to methionine sulfoxide reductase (Msr) enzymes, which are present in all organisms across the tree of life, with few exceptions [7,8]. The two main types are the thiol-oxidoreductases MsrA and MsrB which display strict stereoselectivities toward the *S*- and the *R*-diastereomer of MetO, respectively [9,10]. Genetic and physiological studies revealed important roles of Msrs and of the regulation of MetO content in proteins during oxidative constraints in animals, plants, bacteria, and *Saccharomyces cerevisiae* [11,12,13,14]. Another thiol-oxidoreductase, the fRMsr, is exclusively found in bacteria and unicellular fungi and reduces specifically the free form of Met-*R*-O [15,16].

Bacteria can synthetize several types of molybdenum-containing enzyme families, among which some members of the sulfite oxidase and the dimethyl sulfoxide (DMSO) reductase families were shown to reduce the MetO [17,18]. The sulfite oxidase enzymes possess a molybdenum cofactor in which the Mo atom is coordinated to a dithiolene group of a pterin called molybdopterin [18]. By contrast, the enzymes belonging to the DMSO reductase family have a more complex catalytic center constituted of two molybdopterin guanosine dinucleotides coordinating the Mo atom [18]. The periplasmic methionine sulfoxide reductase (MsrP) belongs to the sulfite oxidase family and was recently shown to be a non-stereospecific protein-MetO reductase with a broad range of protein substrates [19,20]. MsrP is present in most Gram-negative bacteria and genetic characterizations in *Escherichia coli* and *Azospira suillum* indicated that it is principally involved in protein oxidation protection during stress induced by reactive chlorine species [19,21]. The “true” DMSO reductases (i.e., the enzymes which actually reduce the DMSO and serve as prototypes for the DMSO reductase molybdoenzymes family) can be classified into two types, Dms- and Dor-types, with *E. coli* DmsA and *Rhodobacter sphaeroides* DorA, as representative examples, respectively. Note that *R. sphaeroides* DorA could be named DmsA in the literature. DmsA is a trimeric membrane-bound enzyme whereas DorA is a monomeric periplasmic enzyme associated to membrane subunits DorB and DorC [22,23]. Both enzymatic complexes serve as terminal reductases in the anaerobic respiration of DMSO and received electrons from menaquinols or ubiquinols [24]. Of note, in *R. sphaeroides*, DorABC complex is also present during anaerobic photosynthesis and microaerophilic conditions [25]. Substrate specificity characterizations demonstrated that both types of DMSO reductases catalyze the reduction of free MetO and DMSO with similar efficiencies [26,27]. Interestingly, Dms- and Dor-types DMSO reductases have opposite stereospecificities towards the MetO. The *E. coli* DmsA reduces the Met-*R*-O whereas the *R. sphaeroides* DorA and other related enzymes catalyze the reduction of the *S*-diastereomer of MetO [28,29,30,31]. This strict stereospecificity was shown also to be conserved on numerous other sulfoxide containing molecules [28,29,31]. Moreover, two other members of the DMSO reductase molybdoenzyme family, the biotin sulfoxide reductase BisC and the trimethyl N-oxide (TMAO) reductase TorZ/MtsZ, were shown to reduce the free form of Met-*S*-O in the cytosol and the periplasm of some bacteria, respectively [32,33].

The MsrA, MsrB, and MsrP are the only enzymes for which the ability to reduced MetO in oxidized protein was experimentally demonstrated [9,10,19,20]. On the contrary, biochemical assays demonstrated that neither the fRMsr, BisC, nor TorZ/MtsZ can catalyze the reduction of MetO in proteins [15,16,32,33]. The capacity of DMSO reductases to reduce protein bound MetO was not previously evaluated. However, the use of a molecule mimicking a MetO in a peptidic environment suggested that *E. coli* DmsA might reduce oxidized proteins and protect periplasmic proteins from oxidation [30]. But, previous comparisons of Msr activity using oxidized proteins or molecules mimicking peptide bound MetO as substrates revealed that such molecules might not be appropriate model substrates to decipher whether a MetO reductase can or cannot efficiently act on oxidized protein [14]. In this work, we evaluated the capacity of the *R. sphaeroides* DorA DMSO reductase to reduce model oxidized proteins and MetO-containing peptides, and compared it to its ability to reduce DMSO and the free L-Met-*R,S*-O. The first oxidized protein tested was the bovine β-casein, an intrinsically disordered protein possessing 6 Met oxidizable as either Met-*R*-O or Met-*S*-O upon reaction with hydrogen peroxide and previously used for the characterization of several Msr enzymes [14,20]. We also showed that DorA can reduce MetO in two *R. sphaeroides* periplasmic proteins, TakP and PCu_A_C, both potentially exposed to oxidative conditions in the bacteria. TakP is a soluble α-keto acid binding component of a tripartite ATP-independent periplasmic transporter (TRAP) [34]. It contains 11 Met and was found among potential MsrP substrate by proteomics [20]. PCu_A_C is a copper chaperone participating to the metalation of the respiratory cytochrome c oxidase [35]. Its mature form possesses 8 Met, two of which belong to the conserved copper binding motif Hx_n_Mx_22_HxM [36]. Using complementary, enzymatic and mass spectrometric approaches, we showed that DorA can reduce MetO included in these proteins and that it catalyzes the reduction of the oxidized β-casein with a catalytic efficiency comparable to previously characterized Msrs.

## 2. Materials and Methods

### 2.1. Cloning, Protein Expression and Purification

The sequence coding for the full length DorA DMSO reductase (Uniprot accession: Q57366) was amplified from *R. sphaeroides* genomic DNA and cloned into the pIND4 vector [37] using restriction/ligation method to obtain the pIND4-DorA-His plasmid, allowing the expression of the DorA protein with a C-terminal 6-His tag. The sequence coding for mature PCu_A_C (Uniprot accession: Q3J4W6) starting from Ser-23 was amplified from *R. sphaeroides* genomic DNA using primers allowing the inclusion of a 6-His tag and a sequence coding for a Tobacco Etch Virus (TEV) protease site on the N-terminal. The PCR product was ligated in the pET15b vector to obtain the expression plasmid pET15b-6His-TEV-PCu_A_C. All constructs were validated by DNA sequencing.

For the production and the purification of DorA, the pIND4-DorA-His plasmid was incorporated into the *R. sphaeroides* f sp. *denitrificans* IL106 *dorA*^−^ strain [38] by conjugation. The strain was grown in 6-l culture under semi-aerobic conditions in Hutner medium until late exponential phase. The periplasmic fraction was extracted and loaded on a HisTrap column (GE Healthcare, Velizy-Villacoublay, France) using an AKTA^®^ purifier FPLC (GE Healthcare). Then DorA was eluted with 250 mM imidazole in 20 mM 4-(2-hydroxyethyl)-1-piperazineethanesulfonic acid (HEPES), pH 8.0. Elution was followed by measuring absorbance at 280 nm. After concentration using 50-kDa cutoff Amicon^®^ Ultra concentrator (Millipore, Molsheim, France) and buffer exchange to 30 mM Tris-Cl, pH 7.5 using Sephadex G-25 in PD-10 Desalting Columns (GE Healthcare), the protein solution was loaded onto a MonoQ™ 4.6/100 PE (GE Healthcare), and the elution was done with a linear NaCl gradient (0–500 mM). Main fractions were pooled and loaded on a Superdex™ 200 10/30 gel filtration column (GE Healthcare) equilibrated with 30 mM Tris-HCl pH 7.5. The fractions were analyzed by SDS-PAGE and those containing the protein were pooled and concentrated.

For the production and the purification of PCu_A_C, *E. coli* BL21 strain (New England Biolabs, Evry, France) was transformed with the pET15b-6His-TEV-PCu_A_C vector and grown in plate containing lysogeny broth (LB) media with ampicillin (50 µg·mL^−1^). A single colony was used to prepare a 200-mL pre-culture. Then, 6 × 1 L of LB with ampicillin were inoculated with 6 × 25 mL and incubated overnight at 37 °C with horizontal shaking. Cells were harvested by centrifugation at 5000× *g* for 20 min at 4 °C and the pellets were resuspended in 30 mM Tris-Cl, 250 mM NaCl, 25 mM imidazole, pH 7.5 (buffer A) and stored at −80 °C until further use. Cell suspensions were passed twice through a French press at 12,000 psi, and centrifuged at 150,000× *g* for 45 min. The supernatant was applied onto a 5-mL HisTrap column (GE Healthcare) previously equilibrated with buffer A. After washing the column with buffer A, PCu_A_C was eluted with 250 mM imidazole in buffer A. PCu_A_C solution was concentrated using 10-kDa cutoff Amicon^®^ concentrator and desalted with Sephadex G-25 in PD-10 Desalting Columns. The protein concentration was adjusted to 1 mg·mL^−1^ in 30 mM Tris-Cl pH 7.5, 250 mM NaCl, then the TEV protease was added (1:50 TEV:PCu_A_C mass ratio) with 0.5 mM Ethylenediaminetetraacetic acid (EDTA) and 1 mM dithiothreitol (DTT). The solution was incubated overnight at room temperature to remove the polyhistidine tag. TakP (Uniprot accession: Q3J1R2) was produced and purified as described previously [34].

Protein concentrations were determined spectrophotometrically using Pierce™ Coomassie Plus (Thermofisher, Les Ulis, France) and using specific molar extinction coefficients at 280 nm: DorA, 141,200 M^−1^·cm^−1^; TakP, 82,070 M^−1^·cm^−1^; PCu_A_C, 1490 M^−1^·cm^−1^; bovine β-casein (Sigma-Aldrich, Saint-Quentin-Fallavier, France), 11,460 M^−1^·cm^−1.^ Protein solutions were stored at −20 °C until further use. Protein purity was verified using SDS-PAGE gels stained with Expedeon InstantBlue^®^ Protein Stain (VWR, Fontenay-sous-Bois, France).

### 2.2. Protein Oxidation and Peptides

Bovine β-casein (Uniprot accession: P02666) was oxidized with H_2_O_2_ as described previously [20]. TakP (100 μM) in 50 mM sodium citrate pH 5.0 and 100 mM NaCl was incubated with H_2_O_2_ (100 mM i.e., 1,000 molar equivalent) for 18 h at room temperature. H_2_O_2_ was removed by desalting using PD-10 column and the protein solution was concentrated with 10-kDa cutoff Amicon^®^ Ultra concentrator. PCu_A_C (138 µM) in 50 mM 3-morpholinopropane-1-sulfonic acid (MOPS), 150 mM NaCl, pH 7.5 buffer was incubated with H_2_O_2_ (138 mM, i.e., 1,000 molar equivalent) in 1,500-µL reaction for 1 h at room temperature. Then, 1 mL of 1 M DTT solution was added. Protein solution was desalted using PD MiniTrap G-10 (GE Healthcare) in 50 mM MOPS, 150 mM NaCl, pH 7.5, and concentrated. QWGAGM(O)QAEED and TTPGYM(O)EEWNK peptides were obtained from GenScript^®^ (Hong-Kong). Stock solutions were prepared at 20 mM in Britton–Robinson buffer, pH 6.0.

### 2.3. Enzymatic Activity

DorA activity was measured similarly to described in [20] with a few modifications. Benzyl viologen (BV) was used as an electron donor and its consumption was followed at 600 nm using an UVmc1^®^ spectrophotometer (SAFAS, Monaco) equipped with optic fibers in a glovebox workstation (MBRAUN Labstar, Garching, Germany) flushed with nitrogen. Each reaction mixture was made in Britton–Robinson buffer (500-µL final volume). The pH of the solution was 6.0 in most experiments. BV (0.2 mM) and sulfoxide containing substrates were added first in buffer. Then, sodium dithionite was added at 0.8 mM to reduce the BV from the colorless oxidized form to the reduced radical form, to obtain a starting value of absorbance at 600 nm of approximately 1.0, corresponding to 0.1 mM reduced BV. Correction were eventually made with the addition of small amounts of sodium dithionite. The stability of the absorbance at 600 nm was checked for at least one minute. Then, reactions were started by the addition of DorA (23 or 46 nM), and the initial decrease rates of absorbance at 600 nm were measured. Substrate concentrations are indicated in tables and figures legends. We determined that the specific molar extinction coefficients of BV were 8000 M^−1^·cm^−1^ at pH 5.0; 9500 M^−1^·cm^−1^ at pH 5.5; 10,100 M^−1^·cm^−1^ at pH 6.0 and 10,200 M^−1^·cm^−1^ at pH 7.0, in Britton–Robinson buffer. Reduction of MetO rates were calculated from the ΔA_600 nm_ slopes respecting a stoichiometry of 2 (2 moles of BV are oxidized for 1 mole of MetO or DMSO reduced). Thus, the activity values presented as v/[E] or *k*_cat_ (s^−1^) represent the number of moles of MetO reduced per mole of enzyme per second.

### 2.4. Electrospray Ionization-Mass Spectrometry (ESI-MS) Analysis of Purified Proteins

Oxidized β-casein (100 µM), oxidized PCu_A_C (100 µM) and oxidized TakP (50 µM) were reduced by addition of DorA (46 nM) in Britton–Robinson buffer, pH 6.0. BV was added to 0.2 mM and was reduced sequentially with 5 additions of 1.6 mM sodium dithionite every ~20 min. After two hours of reaction in a glove-box, the proteins solutions were desalted, concentrated and stored at −20 °C until analysis. For TakP, oxidized TakP and oxidized β-casein negative controls were made similarly without DorA. ESI-MS analyses were performed on a MicroTOF-Q Bruker (Wissembourg, France) with an electrospray ionization source as described [20].

### 2.5. Search for DorA-Like Proteins

DorA-like protein were searched using NCBI Blastp suite (https://blast.ncbi.nlm.nih.gov/Blast.cgi?PROGRAM=blastp&PAGE_TYPE=BlastSearch&LINK_LOC=blasthome) with *R. sphaeroides* DorA (Uniprot accession: Q3IXS0) as query. Searches were initially made on all the nr database, refined for each bacterial phylum, then on each order. Only proteins possessing the conserved motif ^155^SYGW^158^ shown to be necessary for the reduction of sulfoxides [26] were kept. Partial and multispecies sequences were removed.

## 3. Results and Discussion

### 3.1. The DorA Reductase Reduces MetO in the Oxidized β-Casein with an Efficiency Similar to the Free L-MetO

After the production and the purification of the recombinant DorA, we measured its enzymatic activity on DMSO and L-Met-*R,S*-O to evaluate its efficiency and to determine the optimal parameters to use for the oxidized proteins reduction assays. We used the standard in vitro procedure in which the benzyl viologen (BV), reduced with dithionite, serves as an artificial electron donor for DorA and its oxidation rate is followed spectrophotometrically at 600 nm [17,20,25,39]. We evaluated the pH-dependence of DorA activity between 5.0 and 7.0 (Figure S1). At equal concentration of substrate (1 mM), we observed very similar values of enzyme activity (v/[E], initial rate/total enzyme concentration) using both DMSO and L-Met-*R,S*-O substrates, with maximal values of ~9 s^−1^ at pH 5.0, and values twice lower for higher pHs (Figure S1). This indicates that DorA has a higher activity at acidic pH, as previously observed [40]. Then, for all the following experiments, we chose a pH of 6.0 because of the low solubility of the tested oxidized proteins at lower pH and to prevent PCu_A_C and TakP potential unfolding. To determine the ability of DorA to reduce oxidized protein, we determined the kinetics parameters of the reaction using the oxidized β-casein as substrate, and compared it to those obtained using DMSO and free L-Met-*R,S*-O (Table 1 and Figure S2). With the low molecular weight sulfoxides, we measured similar catalytic constants values (*k_cat_*) for both (~4 s^−1^). The Michaelis-Menten constants (*K_M_*) were ~2 and 70 µM for DMSO and L-Met-*R,S*-O, respectively (Table 1). This resulted in catalytic efficiencies (*k_cat_/K_M_*) values of ~2,200,000 and ~61,000 M^−1^·s^−1^ for DMSO and free L-Met-*R,S*-O, respectively (Table 1). These values were in the range of previously reported data [16,25], and clearly indicated that the purified recombinant DorA was fully functional to evaluate its ability to reduce oxidized proteins. Of note, in parallel of the production of the His-tagged DorA, we made a classical purification procedure to obtain the native enzyme, without any tag, from *R. sphaeroides*. The enzyme was active using both DMSO and free L-Met-*R,S*-O as substrate, but the activity values were around 8 to 10-fold lower than those obtained with the His-tagged DorA (data not shown). Thus, we used the His-tagged DorA for the following experiments. When we used increasing concentrations of oxidized β-casein as substrate, we clearly measured DorA activity and the obtained curve had the typical shape of a Michaelis–Menten kinetics (Table 1 and Figure S2). This first result shows that DorA can reduced the oxidized β-casein. We determined a catalytic constant value similar to those obtained with the low molecular weight sulfoxide (*k_cat_* ~1.6 s^−1^) and also a low *K_M_* (~20 µM), giving an apparent catalytic efficiency of ~90,000 M^−1^·s^−1^ (Table 1). These results confirmed that DMSO is very likely the preferred substrate for DorA, at least in vitro, but indicated that the enzyme can catalyze the reduction of an oxidized protein with an efficiency similar to the reduction of the free oxidized amino acid. However, for an accurate comparison with the other tested substrates, the *K_M_* value should be corrected by the number of MetO reduced by DorA in the oxidized protein. Indeed, the β-casein possesses 6 Met and previous characterization showed that they were all oxidizable as racemic mixture of *R*- and *S*-diastereomer of MetO, but the MetO formed were not equivalent substrates for the other Msrs [14,20].

**Figure 1:**
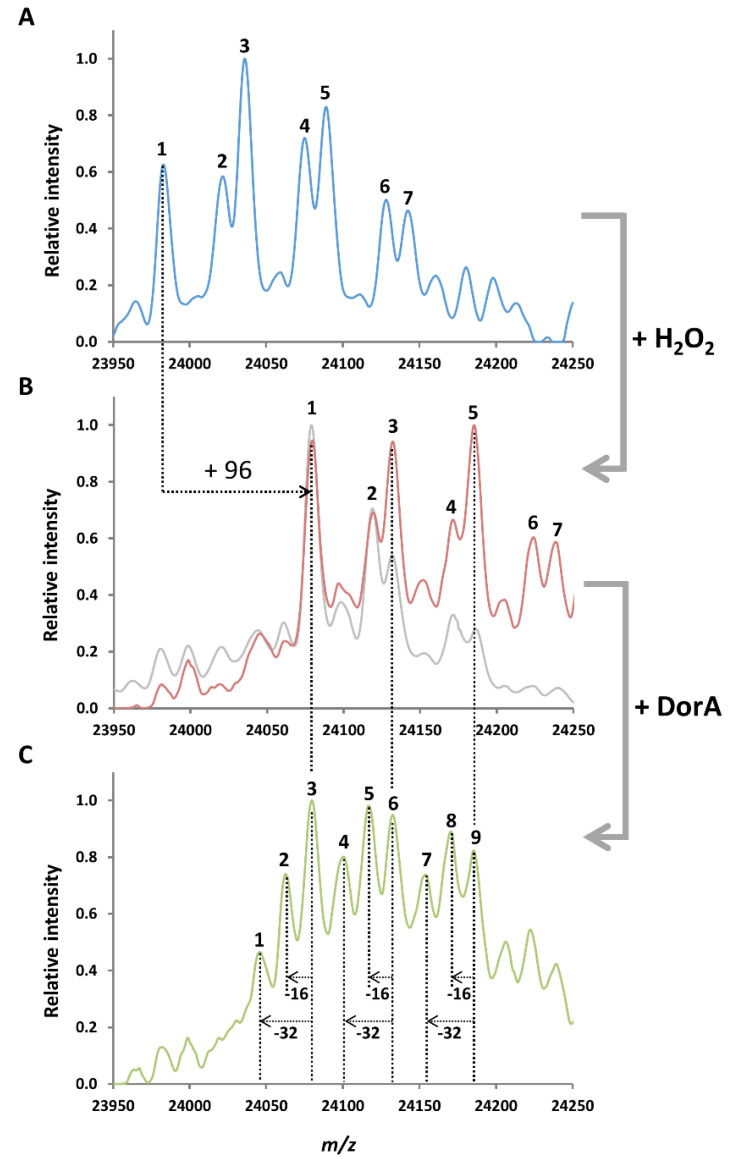
Oxidation of β-casein by H_2_O_2_ and reduction by DorA analyzed by ESI-MS. (**A**) Commercial β-casein exists as a mixture of genetic variants (7 main in this batch). β-casein was analyzed by ESI–MS. Main peak masses: 1. 23,982.7 Da; 2. 24,021.6 Da; 3. 24,035.0 Da; 4. 24,075.0 Da; 5. 24,089.1 Da; 6. 24,127.5 Da; 7. 24,142.5 Da. (**B**) β-casein was oxidized with 50 mM H_2_O_2_ before MS analysis. All major peaks underwent an increase in ~96 Da compared with the non-oxidized sample. The red spectrum corresponds to the oxidized β-casein directly analyzed by ESI-MS whereas the grey spectrum corresponds to the oxidized β-casein incubated with BV and dithionite, but in absence of DorA. This negative control showed that no reduction is observed in absence of DorA. Main peaks masses: 1. 24,080.6 Da; 2. 24,120.2 Da; 3. 24,132.7 Da; 4. 24,172.7 Da; 5. 24,185.2 Da; 6. 24,224.8 Da; 7. 24,239,6 Da. (**C**) Oxidized β-casein was incubated with DorA (46 nM) in the presence of BV (0.2 mM) and sodium dithionite (8 mM) as electron donors before MS analysis. Main peaks masses: 1. 24,046.9 Da; 2. 24,063.5 Da; 3. 24,080.4 Da; 4. 24,101.7 Da; 5. 24,117.9 Da; 6. 24,133.5 Da; 7. 24,155.2 Da; 8. 24,171.3 Da; 9. 24,186.0 Da.

To estimate the number of MetO reduced by DorA in the oxidized β-casein, we determined the total mass of the protein before and after hydrogen peroxide oxidation, and after incubation with DorA using ESI-MS analysis (Figure 1). Oxidation and reduction of Met should respectively result in increases and decreases in mass equal to a multiple of 16 (the mass of the oxygen atom added on the Met). The H_2_O_2_ treatment led to the complete oxidation of the 7 variants present in the commercial mixture with mass increases of 96 Da, corresponding to the oxidation of the 6 Met present in the protein (Figure 1A,B). Previous characterization showed that this oxidative treatment led to the formation of no other oxidative modifications than the 6 MetO as the incubation with the non-stereospecific protein bound MetO reductase MsrP restored the initial mass of the β-casein [20]. Incubation of the oxidized β-casein with DorA led to the apparition of peaks with lower masses (Figure 1C). Principally, focusing on the 3 main variants of oxidized β-casein (peaks 1, 3, and 5 in Figure 1B), we observed 3 corresponding peaks after the action of DorA for each variant: a peak with an unchanged mass (peaks 3, 6, and 9 in Figure 1C), a peak with a decreased mass of 16 Da (peaks 2, 5 and 8 in Figure 1C) and a peak with a mass decrease of 32 Da (peaks 1, 4 and 7 in Figure 1C). This analysis indicates that DorA can reduce 1 or 2 MetO in the oxidized β-casein in the tested conditions (Figure 1C). It could be noted that the treatment of the oxidized β-casein by the reducing system (benzyl viologen and dithionite) in absence of DorA did not induced the apparition of peaks with lowered mass, and thus, that the reduction of the oxidized β-casein was specific of DorA (Figure 1B). The fact that DorA did not reduced a higher number of MetO could be due to its stereospecificity, at least partially. Indeed, DorA was shown to reduce stereospecifically the *S*-diastereomers of low molecular weight sulfoxide containing molecules [28,29], and this stereospecificity is very likely conserved in oxidized proteins, although it remains to be experimentally validated. This is in agreement with previous results showing that the yeast MsrA, also stereospecific towards the *S*-diastereomer of MetO, reduces an average of ~2.5 MetO in the oxidized β-casein [14]. In addition to confirm that DorA reduced MetO in this oxidized protein model, it also allowed us to correct the *K_M_* value obtained previously (Table 1). Indeed, as DorA can reduce up to 2 MetO in the oxidized β-casein, the *K_M_* should be multiplied by 2 as 1 mole of oxidized β-casein corresponded to 2 moles of substrate for DorA (Table 1). Thus, the correction gave a *K_M_* value of ~37 µM and a corresponding catalytic efficiency of ~45,000 M^−1^·s^−1^. These values remain similar to those obtained using the free MetO as substrate. The kinetic parameters obtained for the yeast MsrA and MsrB with the oxidized β-casein as substrate, were very similar, with *k_cat_* and *K_M_* around 1 s^−^^1^ and 50 µM, respectively [14]. However, as the reducing systems were different (NADPH and thioredoxin system for Msrs vs. dithionite and BV for DorA), this comparison should be considered carefully. On the other hand, the kinetic parameters determined with the *R. sphaeroides* MsrP on an oxidized β-casein containing only the *S*-diastereomer of MetO, and using the same reducing system than for DorA, were also very similar (*k_cat_* ~8 s^−^^1^, *K_M_* ~50 µM and *k_cat_/K_M_* ~140,000 M^−1^·s^−1^) [20]. Altogether, these enzymatic assays and mass spectrometry experiments demonstrate that the *R. sphaeroides* DorA reductase can catalyze the reduction of MetO in the oxidized β-casein with a catalytic efficiency similar to the other well-known Msrs.

### 3.2. DorA Reduces MetO in Two Physiologically Relevant Periplasmic Oxidized Proteins

**Figure 2:**
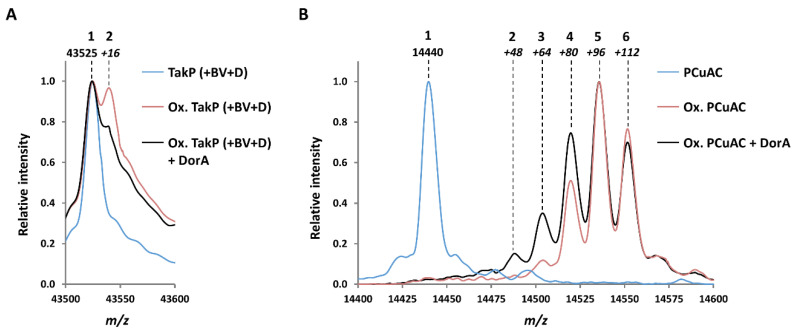
Oxidation of the *R. sphaeroides* TakP and PCu_A_C periplasmic proteins by H_2_O_2_ and reduction by DorA analyzed by ESI-MS. (**A**) Untreated TakP (blue curve ‘TakP (+BV+D)’), TakP oxidized with H_2_O_2_ (red curve ‘Ox. TakP (+BV+D)’) and oxidized TakP incubated with DorA DMSO reductase (black curve ‘Ox. TakP (+BV+D) + DorA’). Note that TakP and the oxidized TakP were incubated with BV and dithionite (D), but in absence of DorA, in the same conditions used for the reduction by DorA. Main peaks masses: 1. 43,524.8 Da; 2. 43,541.1 Da. (**B**) Untreated PCu_A_C (blue curve ‘PCu_A_C’), PCu_A_C oxidized with H_2_O_2_ (red curve ‘Ox. PCu_A_C’) and oxidized PCu_A_C incubated with DorA DMSO reductase (black curve ‘Ox. PCu_A_C + DorA’) were analyzed by ESI-MS. Main peaks masses: 1. 14,439.7 Da; 2. 14,489.7 Da; 3. 14,503.8 Da; 4. 14,519.7 Da; 5. 14,535.8 Da; 6. 14,551.7 Da.

If the β-casein presents the great advantage to be available in high quantity and easily oxidizible, it is a naturally unstructured protein not physiologically relevant for *R. sphaeroides*. Therefore, we wanted to evaluate whether DorA could reduce MetO in structurated proteins, potentially subjected to oxidative pressure in the purple bacteria. We chose two *R. sphaeroides* periplasmic proteins as model: TakP, an α-keto acid binding protein for which we previously evidenced the formation of MetO and its partial reduction by MsrP [20,34] and PCu_A_C, a copper chaperone [36]. Preliminary results indicate that PCu_A_C might be a physiological substrate of MsrP (manuscript in preparation). TakP and PCu_A_C possess 11 and 8 Met, respectively. To oxidize them, we treated both proteins with 1,000 molar equivalents of hydrogen peroxide. In the case of TakP, the ESI-MS analysis showed that the protein oxidation was incomplete (see after, Figure 2A), but oxidation assays with higher H_2_O_2_ concentrations induced the precipitation of the protein. For PCu_A_C, the oxidation was more efficient (see after, Figure 2B) but the oxidized protein was rapidly degraded after the treatment. Because of this sensibility to the oxidative treatment and the difficulty to purify high amount of proteins, we could not determine the kinetics parameters of the reduction reaction as described for the oxidized β-casein (Table 1). Hence, we evaluated the ability of DorA to reduced MetO in these proteins by determining their total masses by ESI-MS analysis (Figure 2). The analysis of the untreated TakP indicated that it has a mass of 43,525 Da (peak 1 in Figure 2A), corresponding to the expected value based on the primary sequence. After oxidation, we observed two peaks corresponding to masses of 43,525 Da and 43,541 Da (peaks 1 and 2 in Figure 2A). The first peak had a mass equivalent of the non-oxidized protein, and the second peak, having an increased mass of 16 Da by comparison with the first, corresponded very likely to an oxidized TakP having one MetO only. We incubated the oxidized TakP with DorA and observed an important, but incomplete, decrease of the relative intensity of the second peak (peak 2 in Figure 2A), indicating that DorA can reduce the oxidized TakP, at least partially. A similar ESI-MS analysis of the untreated PCu_A_C protein indicated that it has a mass of 14,440 Da (Figure 2B)**,** as expected from the primary sequence. The analysis of the protein after oxidation with H_2_O_2_ led to the apparition of 4 peaks with mass increases of 64, 80, 96, and 112 Da, corresponding to 4, 5, 6, and 7 oxidized Met, respectively. As PCu_A_C possesses 8 Met (but no Cys), this result indicates that the oxidation of the protein was not complete. The incubation with DorA led to a limited reduction of the oxidized protein, with higher proportion of the peaks corresponding to the protein with 4 and 5 MetO (peaks 3 and 4 in Figure 2B), compared to the oxidized protein. This also led to the apparition of a peak with a mass corresponding to 3 MetO (peak 2, +48 Da, compared to the untreated protein) (Figure 2B). This result indicates that DorA can reduce the oxidized PCu_A_C protein, but only partially also.

These results showed that the DorA can reduce MetO, not only in the unfolded model β-casein, but also in physiologically relevant periplasmic proteins. However, in the case of both the oxidized TakP and PCu_A_C, the DorA efficiency appeared to be limited. This could be potentially explained by the stereospecificity of DorA, and the proportion of each diastereomer of MetO in the oxidized proteins. Indeed, as previous results indicated that DorA reduces only the Met-*S*-O, it very likely could not reduce the *R*-diastereomers formed in the oxidized proteins. As the proportion of each diastereomer is not inferred from our experiences, it is difficult to determine the reduction efficiency by DorA. Moreover, the partial reduction of these oxidized proteins could also be explained by sequence and structure properties of these substrates precluding efficient catalysis of the MetO reduction, although it was present as *S*-diastereomer. Indeed, in our previous proteomics analysis of the periplasmic oxidized proteins reduced by the non-stereospecific MsrP, we found numerous proteins having MetO not reduced at all or not efficiently reduced by the reductase [20]. This was the case for TakP, for which an oxidized Met was reduced at ~20% only [20]. Similar properties were determined for MsrA and MsrB substrates [14,41,42].

### 3.3. DorA Capacity to Reduce MetO in Protein is Influenced by the Primary Sequence

**Figure 3:**
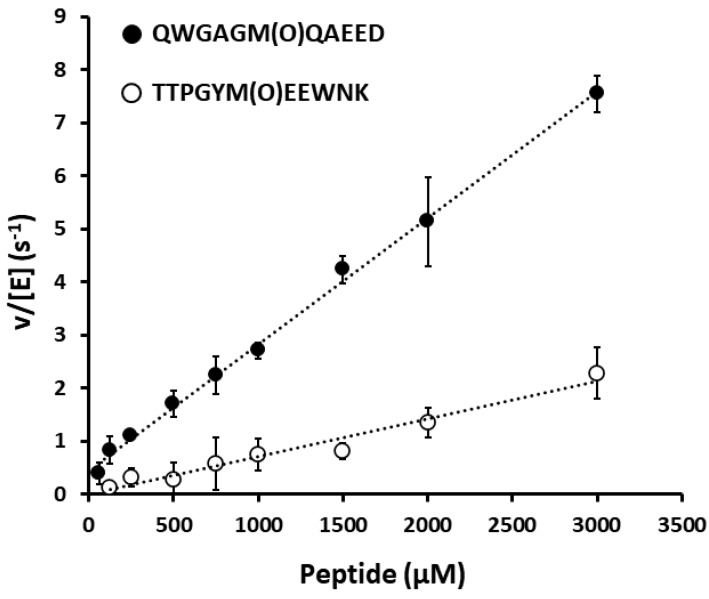
Reductase activity of DorA using the MetO-containing peptides QWGAGM(O)QAEED and TTPGYM(O)EEWNK as substrates. The reductase activity was measured in Britton–Robinson buffer, pH 6.0 with 46 nM DorA and peptides concentrations ranging from 63 to 3000 µM. Data presented are averages of three replicates ± SD.

The previous characterizations of protein bound MetO reductases indicated that their ability to reduce a MetO depends on the sequence properties of the substrate proteins, with the most efficiently reduced MetO generally surrounded by hydrophilic amino acids. This is very likely linked to structural properties because such amino acids are usually present at the surface of proteins, in which MetO are more accessible for an enzymatic reduction [14,20,41,42]. To determine whether or not the DorA capacity to reduce a MetO depends on surrounding amino acid sequence, we tested its reductase activity on two model peptides. The two selected peptides, QWGAGM(O)QAEED and TTPGYM(O)EEWNK, arose from our previous proteomic characterization of the MsrP substrate specificity, and are representative of sequences surrounding MetO efficiently or inefficiently reduced by MsrP, respectively, in periplasmic oxidized proteins [20]. Activity measurements made using increasing concentrations of each peptide clearly showed that DorA can reduce both peptides but acts preferentially on the QWGAGM(O)QAEED peptide (Figure 3). For instance, at the maximal concentration of peptide assayed (3 mM), we measured a ~3-fold higher value with the QWGAGM(O)QAEED peptide than with TTPGYM(O)EEWNK peptide (v/[E] 7.5 ± 0.3 s^−1^ and 2.3 ± 0.5 s^−1^, respectively). These results clearly indicate that the capacity of DorA to reduce a MetO in a proteinaceous substrate is influenced by its primary sequence, similarly to the previously characterized Msrs [14,20,41,42]. Moreover, it is interesting to note that the v/[E] value obtained with the QWGAGM(O)QAEED peptide was 5-fold higher than the *k_cat_* obtained using the oxidized β-casein (see Table 1). We obtained similar results during the characterization of the *R. sphaeroides* MsrP enzyme [20]. Similar differences of *k_cat_* values using several MetO susbtrates were also previously observed for the yeast MsrA and MsrB [14]. This suggests that the nature of amino acids surronding the MetO to be reduced must have a strong influence on the enzyme activity, not only on its apparent affinity but also on its capacity to catalyze the reduction.

## 4. Conclusions

In sum, our work demonstrates that *R. sphaeroides* DorA can reduce MetO in oxidized proteins in vitro, with an efficiency similar to previously characterized Msrs [14,19,20]. Completely unrelated to the thiol oxidoreductases MsrA or MsrB and having a very different Mo-cofactor than MsrP, DorA constitutes thus a potential fourth type of protein-MetO reductase. A comparison of the active site access in all these Msrs shows that they all share a relatively highly surface exposed active site, which is compatible with their function (Figure S3). By contrast, it was shown that the selectivity of fRMsr for free Met-*R*-O is partially due to the enclosed form of its active site, precluding the access of MetO within a peptide [16]. A BLAST search of DorA-like proteins possessing the conserved motif ^155^SYGW^158^ necessary for the reduction of sulfoxides [26] showed that this potential protein-bound MetO reductase is potentially conserved across almost all proteobacteria orders and in some actinobacteria (Supplementary data). Among these, DorA-like proteins are found in aquatic bacteria (Rhodobacteraceae) and potential plant symbiont (Rhizobiaceae and Bradyrhizobiaceae). They are also present in several human pathogens of Enterobacteriaceae, Yersiniaceae and Campylobacteriaceae families such as *Klebsiella pneumoniae*, *Salmonella enterica*, *Shigella flexneri*, *Yersinia pestis,* or *Campylobacter jejuni* (Supplementary data). In these bacteria, the DorA-like enzyme could reduce various types of sulfoxide substrates, from low molecular weight molecules like DMSO, to oxidized peptides and proteins. As most of these bacteria possess also a periplasmic MsrP, the DorA-like could have a complementary role either by being expressed in different physiological conditions, or in conditions where both proteins are present. Particularly, in *R. sphaeroides* these proteins are expressed simultaneously in a wide range of conditions during which oxidant molecules could form *R*- and *S*-diastereomers of MetO on the periplasmic proteins [25,43,44]. As MsrP has a 6-fold less efficiency on Met-*S*-O, DorA could bring a complementary Met*-S*-O reductase activity for the reduction of the same pool of protein substrates or act on different targets. We already knew that in *R. sphaeroides*, DorA had two main roles. First, it allows the growth under dark anaerobic conditions with DMSO respiration [40]. Furthermore, under high reducing conditions, it participates to the oxidation of the quinones pool, restoring efficient cyclic photosynthetic electron transfer. Indeed, when most of the quinones are reduced, the quinones pool is not able to accept electrons from the reaction center anymore and the electron transfer is slowed down [43]. Now, we can add that it also potentially protects proteins from oxidation. DorA could thus use a large set of sulfoxide-containing molecules to fuel up the electron transport chain. Although this function remains to be validated in vivo, the finding the DorA could participate into the protection of the periplasmic proteins against oxidative stress brings new insights into how bacteria cope with a harmful environment.

## Figures and Tables

**Table 1:** Kinetics parameters of DorA towards MetO-containing substrates. Reactions were made in Britton–Robinson buffer at pH 6.0 with 46 nM DorA and DMSO (1.6 to 200 µM), free L-Met-*R*,*S*-O (3.9 to 2,000 µM) or oxidized β-casein (1.6 to 200 µM). Data presented are averages of three replicates ±SD. Saturation curves are presented in Figure S2.

Substrate		*k_cat_* (s^−1^)	*K_M_* (µM)	*k_cat_*/*K_M_* (M^−1^·s^−1^)
DMSO		3.9 ± 0.1	1.8 ± 0.4	2,200,000
*L*-Met-*R,S*-O ^1^		4.2 ± 0.1	68.8 ± 5.7	61,000
Oxidized β-casein	*Measured*	1.6 ± 0.2	18.6 ± 6.0	90,000
	*Corrected* ^2^		37.2 ± 12.0	45,000

^1^ Considering that only the *S*-diastereomer serve as substrates for DorA reductase [28] and assuming that the *R*-diastereomer does not act as inhibitor, the *K_M_* values were divided by 2. ^2^ As the ESI-MS analysis indicated that 2 MetO are available substrate for DorA (Figure 1), the *K_M_* value was multiplied by 2.

## Supplementary Materials

The following are available online at https://www.mdpi.com/2076-3921/9/7/616/s1, Figure S1: pH-dependent activity of DorA using DMSO and free L-Met-*R,S*-O as substrates. Figure S2: Reductase activity of DorA using DMSO, free L-Met-*R,S*-O and oxidized β-casein as substrates. Figure S3: Comparison of the active site accessibility in different Msrs. Supplementary data: DorA-like proteins sequences.
