# Supplementary material for: Reduction of Protein Bound Methionine Sulfoxide by a Periplasmic Dimethyl Sulfoxide Reductase
Source: Antioxidants (Basel). 2020 Jul 14;9(7):616. doi: 10.3390/antiox9070616 (PMC7402097; doi:10.3390/antiox9070616)
Supplement: Supplementary file 1 [file antioxidants-09-00616-s001.zip › Tarrago_Supplementary files/DMSO red_Supp figures 20 05 2020.pdf]

Reduction of protein-bound methionine sulfoxide by a periplasmic dimethyl sulfoxide reductase

Lionel Tarrago[1,2,*], Sandrine Grosse[1], David Lemaire[1], Laetitia Faure[1], Mathilde Tribout[1], Marina I. Siponen[1], Mila Kojadinovic-Sirinelli[1], David Pignol[1], Pascal Arnoux[1], Monique Sabaty[1,*]

[1] Aix Marseille Univ, CEA, CNRS, BIAM, Saint Paul Lez Durance, France F 13108

[2] Biodiversité et Biotechnologie Fongiques, UMR1163, INRAE, Aix Marseille Université, Marseille, France.

*Corresponding authors: Lionel Tarrago (lionel.tarrago@inrae.fr) and Monique Sabaty (monique.sabaty@cea.fr)

Supplementary figures

Supplementary Figure 1. pH-dependent activity of DorA using DMSO and free L-Met-*R,S*-O as substrates. **2**

Supplementary Figure 2. Reductase activity of DorA using DMSO, free L-Met-*R,S*-O and oxidized β-casein as substrates. **3**

Supplementary Figure 3. Comparison of the active site accessibility in different Msrs. **4**

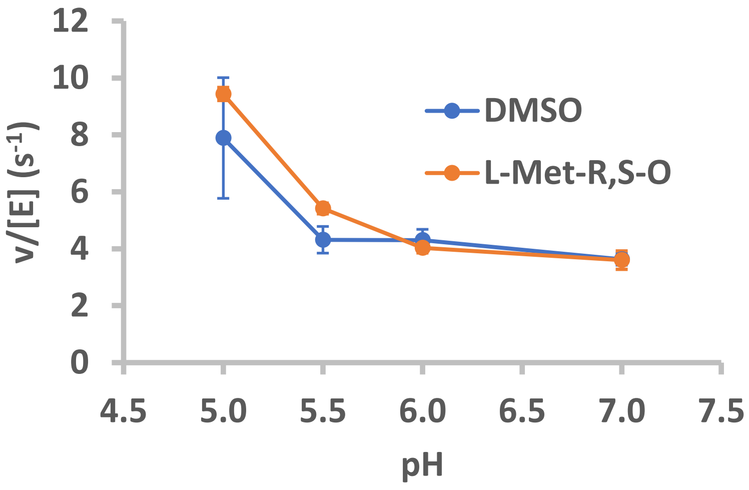

Supplementary Figure 1. pH-dependent activity of DorA using DMSO and free L-Met-*R,S*-O as substrates. DMSO reductase activity was measured as described in *Materials and Methods* in Britton-Robinson buffer at four pHs (5.0; 5.5; 6.0 and 7.0) with 23 nM DorA and 1 mM DMSO or 1 mM free L-Met-*R,S*-O. Data presented are averages of three replicates ± SD.

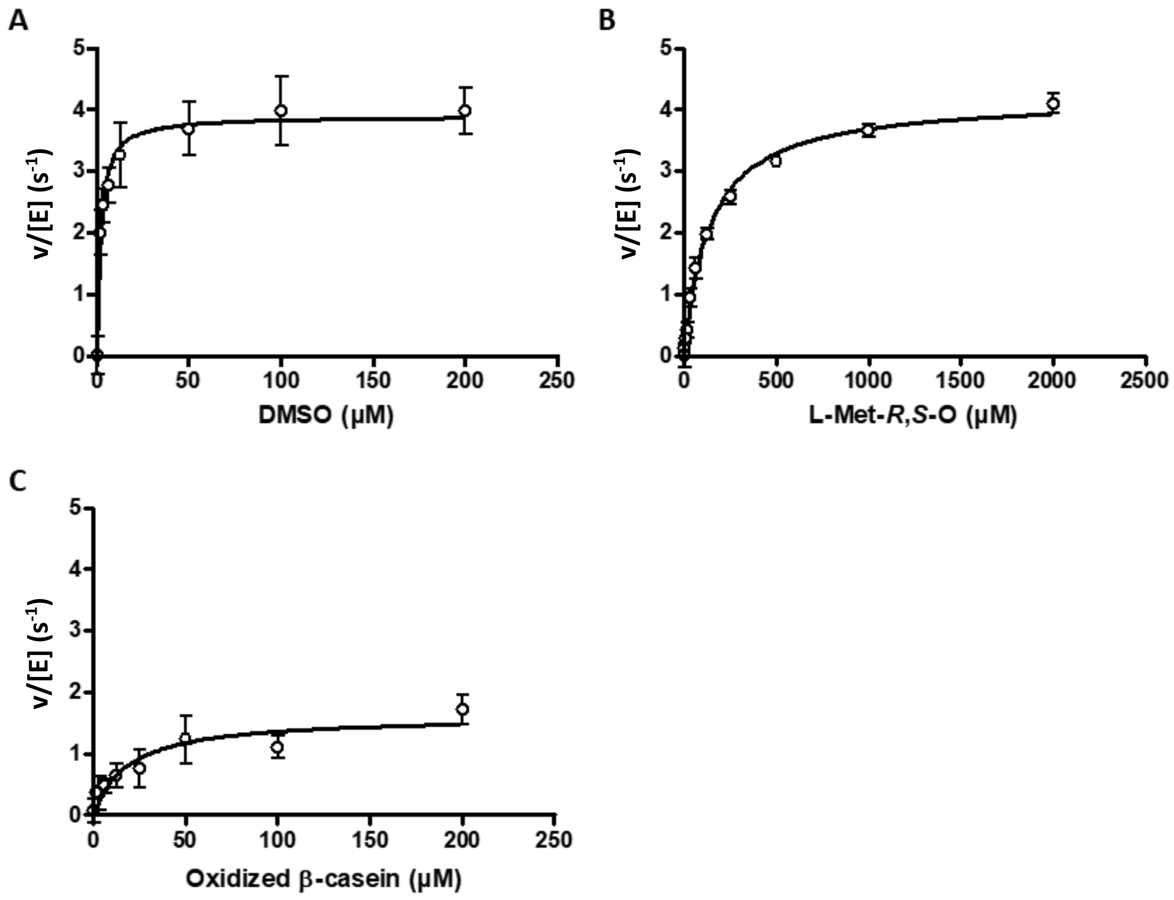

Supplementary Figure 2. Reductase activity of DorA using DMSO (A), free L-Met-*R,S*-O (B) and oxidized β-casein (C) as substrates. Assays were carried out similarly to described in **Fig. 1**. Reaction were made in Britton-Robinson buffer at pH 6.0 with 46 nM DorA and DMSO (1.6 to 200 µM), free L-Met-*R,S*-O (3.9 to 2 000 µM) or oxidized β-casein (1.6 to 200 µM). Data presented are averages of three replicates ± SD.

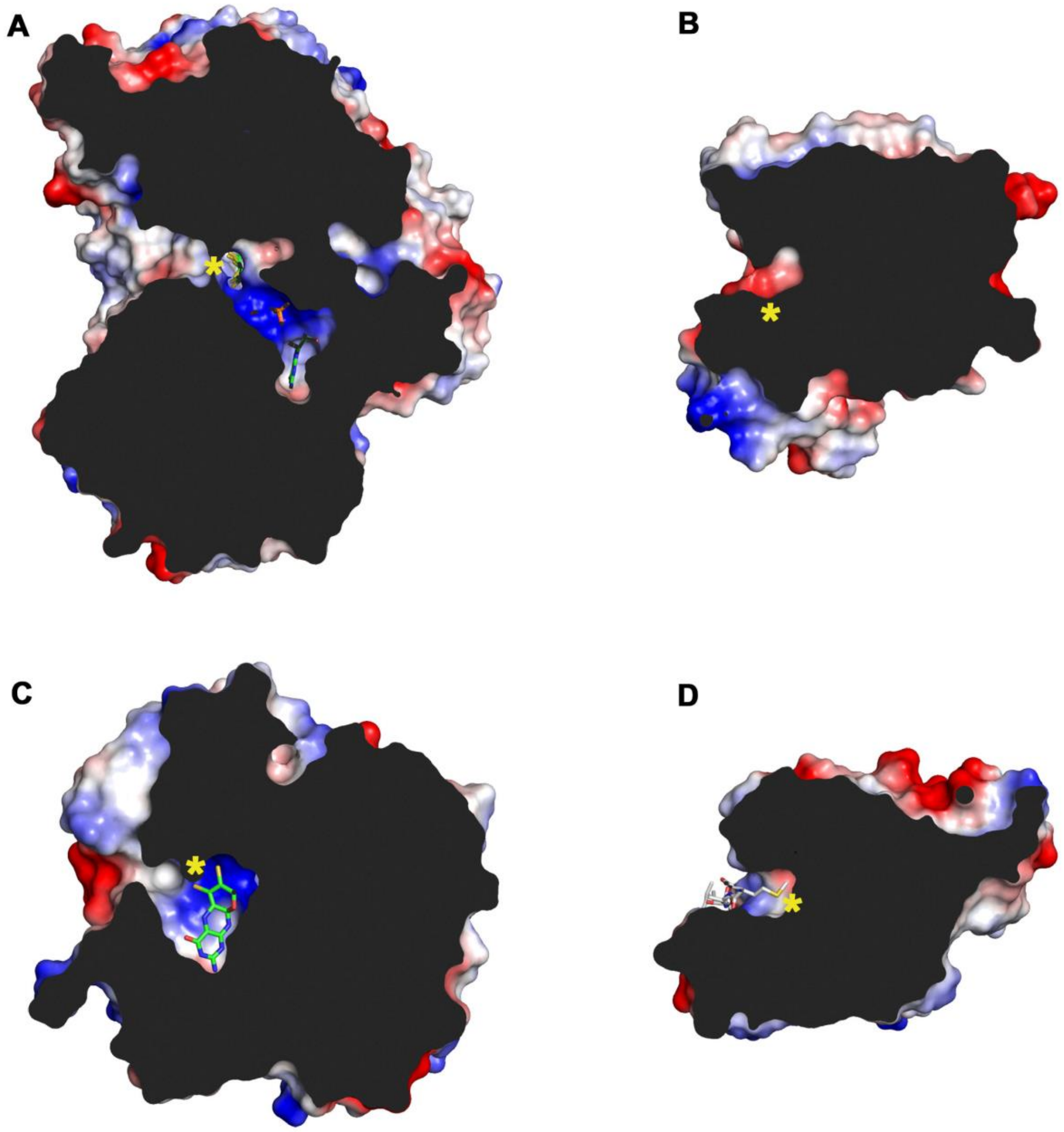

Supplementary Figure 3. Comparison of the active site accessibility in different Msrs. (**A**) *Rhodobacter capsulatus* DMSO reductase (pdb code 1eu1), (**B**) *Escherichia coli* MsrA (pdb code 1ff3), (**C**) *E. coli* MsrP (pdb code 1xdq), (**D**) *Bacillus subtilis* MsrB (in which the MsrA domain was omitted and where the relevant substrate peptide containing a methionine is shown in stick; pdb code 3e0o). In each cases the active site residues (Cys in MsrA and MsrB, Molybdenum in MsrP and DMSO reductase) is marked with a yellow asterisk).
